# Determinants of health after hospital discharge: rationale and design of the Vanderbilt Inpatient Cohort Study (VICS)

**DOI:** 10.1186/1472-6963-14-10

**Published:** 2014-01-08

**Authors:** Abby G Meyers, Amanda Salanitro, Kenneth A Wallston, Courtney Cawthon, Eduard E Vasilevskis, Kathryn M Goggins, Corinne M Davis, Russell L Rothman, Liana D Castel, Katharine M Donato, John F Schnelle, Susan P Bell, Jonathan S Schildcrout, Chandra Y Osborn, Frank E Harrell, Sunil Kripalani

**Affiliations:** 1 Vanderbilt University School of Medicine, Nashville, TN, USA; 2 VA Tennessee Valley Geriatric Research Education Clinical Center (GRECC), HSR&D Targeted Research Enhancement Program for Patient Healthcare Behavior, Nashville, TN, USA; 3 Clinical Research Center of Excellence (CRCoE), Nashville, TN, USA; 4 Division of General Internal Medicine and Public Health, Department of Medicine, Vanderbilt University, 1215 21st Ave S, Suite 6000 Medical Center East, Nashville 37232, TN, USA; 5 School of Nursing, Vanderbilt University, Nashville, TN, USA; 6 Center for Health Services Research, Vanderbilt University, Nashville, TN, USA; 7 Department of Sociology, Vanderbilt University, Nashville, TN, USA; 8 Center for Quality Aging, Vanderbilt University, Nashville, TN, USA; 9 Division of Cardiovascular Medicine, Vanderbilt University, Nashville, TN, USA; 10 Department of Biostatistics, Vanderbilt University, Nashville, TN, USA; 11 Department of Biomedical Informatics, Vanderbilt University, Nashville, TN, USA

**Keywords:** Hospitalization, Patient discharge, Patient readmission, Transitions of care, Health literacy, Social determinants

## Abstract

**Background:**

The period following hospital discharge is a vulnerable time for patients when errors and poorly coordinated care are common. Suboptimal care transitions for patients admitted with cardiovascular conditions can contribute to readmission and other adverse health outcomes. Little research has examined the role of health literacy and other social determinants of health in predicting post-discharge outcomes.

**Methods:**

The Vanderbilt Inpatient Cohort Study (VICS), funded by the National Institutes of Health, is a prospective longitudinal study of 3,000 patients hospitalized with acute coronary syndromes or acute decompensated heart failure. Enrollment began in October 2011 and is planned through October 2015. During hospitalization, a set of validated demographic, cognitive, psychological, social, behavioral, and functional measures are administered, and health status and comorbidities are assessed. Patients are interviewed by phone during the first week after discharge to assess the quality of hospital discharge, communication, and initial medication management. At approximately 30 and 90 days post-discharge, interviewers collect additional data on medication adherence, social support, functional status, quality of life, and health care utilization. Mortality will be determined with up to 3.5 years follow-up. Statistical models will examine hypothesized relationships of health literacy and other social determinants on medication management, functional status, quality of life, utilization, and mortality. In this paper, we describe recruitment, eligibility, follow-up, data collection, and analysis plans for VICS, as well as characteristics of the accruing patient cohort.

**Discussion:**

This research will enhance understanding of how health literacy and other patient factors affect the quality of care transitions and outcomes after hospitalization. Findings will help inform the design of interventions to improve care transitions and post-discharge outcomes.

## Background

### Hospital discharge and poor transitions in care

Research has demonstrated that the period following hospital discharge is a vulnerable time for patients. As patients return home from the hospital, they must often manage new health care problems, changes in their medication regimen, and follow-up appointments, even as they continue to recover from an acute illness [1]. Medication errors, adverse drug events, and hospital readmission are major concerns [1]. For example, the 30-day rehospitalization rate among Medicare beneficiaries is 19.6%, with an estimated annual cost to Medicare of $17.4 billion [2].

A better understanding is needed of which patients are at highest risk for adverse outcomes in this critical time period. Patients with cardiovascular conditions, such as acute coronary syndromes (ACS) or acute decompensated heart failure (ADHF), are a logical choice for study, as they are frequently required to manage a complex set of medications and other self-care activities after hospital discharge. In this context, suboptimal self-care, such as medication non-adherence, is common and is associated with recurrent cardiac events, re-hospitalization, and higher mortality [3]. Overall, the 30-day incidence of readmission following ACS is about 15%; [4] the 1-year mortality in registries observing a broad spectrum of ACS patients is 2.3% to 8.6% [5,6]. For ADHF, the 30-day readmission rate is approximately 23%, [7] and 1-year mortality after hospitalization is about 27% [8].

### Predictors of post-discharge outcomes

Most studies that have examined predictors of post-discharge outcomes have looked only at variables that are readily available in administrative datasets [9]. Such work has shown, for example, that African Americans, older adults, and individuals in lower socioeconomic strata experience higher readmission rates [2]. Large ongoing observational studies are focusing on racial and gender disparities in post-hospitalization outcomes for patients with acute myocardial infarction [10,11]. Yet, relatively few studies have examined how complex and interrelated social factors may affect readmission and mortality after hospitalization [12,13].

Social determinants of health, which account for some health disparities, [14] include measures of sociodemographic, educational, cognitive, psychological, cultural, and behavioral variables [15]. Predictive models that include social determinants are better at forecasting readmission than models solely based on administrative data [9]. Health literacy is an important social determinant which affects a patient’s ability to appropriately take medications, keep follow-up appointments, watch for signs of worsening illness, and know what to do if they occur [16]. Low health literacy is independently associated with hospital readmission, [17] as well as mortality [18]. Social support from friends or family is another important factor in the post-discharge period [12]. Such patient-level factors, as well as system-level factors like timeliness of post-discharge follow-up and quality of medication reconciliation, likely play a large role in post-discharge outcomes, but more in-depth measurement and analysis of such factors is needed to determine their relative contributions and interrelationships [12].

In addition to informing risk prediction, a better understanding of determinants of health after hospitalization will inform intervention delivery. Many studies have examined the efficacy of interventions to improve care transitions and reduce hospital readmission, some tailored to a patient’s level of health literacy and social circumstances [19]. Most interventions are deployed across broad patient populations, though some studies identify high-risk patients for intervention, on the basis of medical or psychiatric conditions, functional status, previous admissions, age, or other factors. However, what characteristics define a high-risk state varies across studies. Improved ability to identify high-risk patients through more robust assessment of their social determinants would facilitate more targeted, efficient, and cost-effective delivery of transitional care interventions [20].

**Figure 1:**
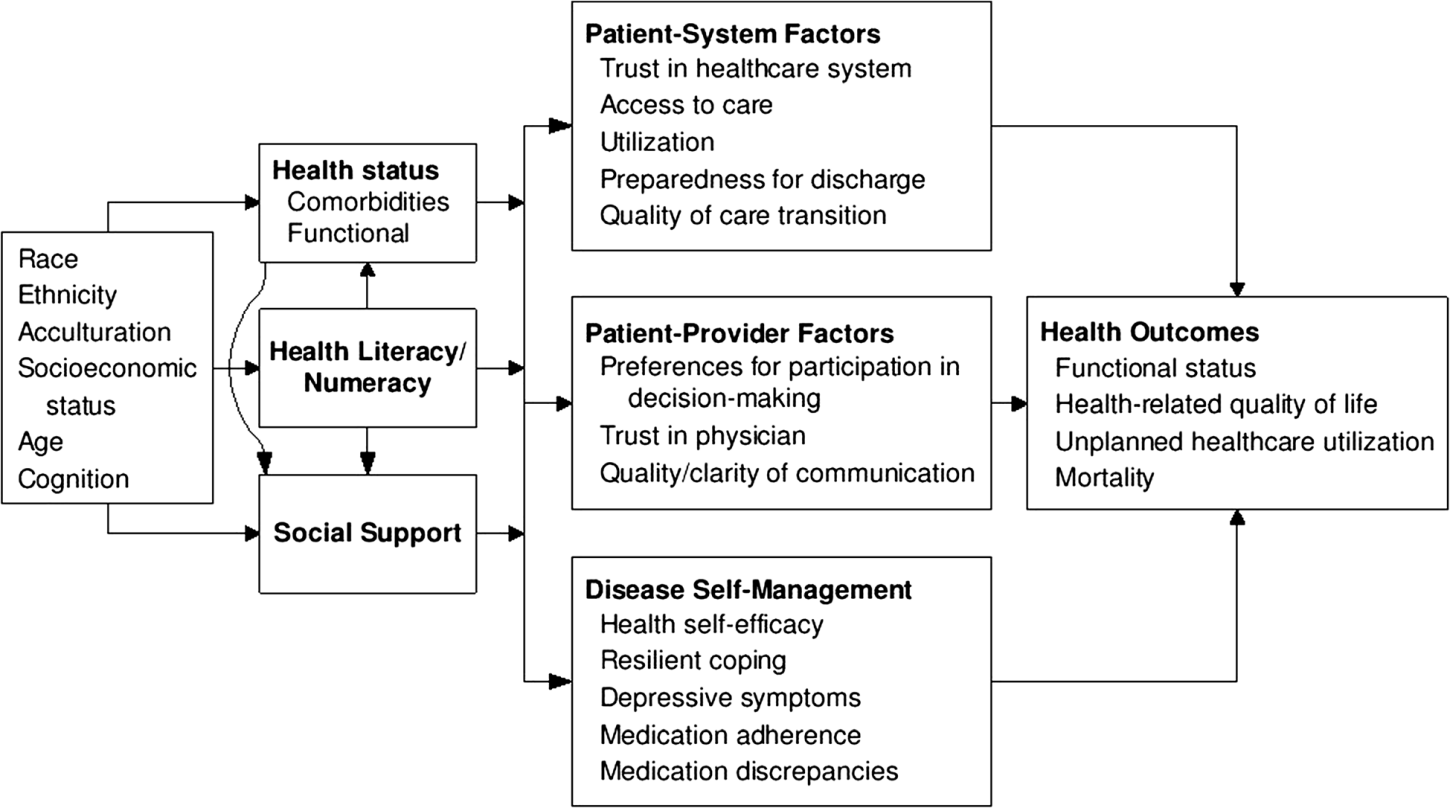
Framework relating patient characteristics to health outcomes.

We delineate a model to guide further study of social determinants of health after hospital discharge (Figure 1), which is based in part on a prior model relating health literacy and self-care to health outcomes [21]. The figure displays (from left to right) demographic attributes such as age, race, ethnicity, and socioeconomic standing that influence health status, social support, and health literacy. In turn, these three aspects affect how patients interact with the healthcare system, the healthcare providers, and the ability to manage their disease(s). Ultimately, all factors impact health outcomes of interest, including functional status, health-related quality of life, unplanned healthcare utilization, and mortality. (For clarity, not all posited relationships are marked with arrows. For example, socioeconomic status may directly affect health care utilization).

### Aims of the Vanderbilt Inpatient Cohort Study (VICS)

The Vanderbilt Inpatient Cohort Study (VICS) is a 5-year prospective longitudinal cohort study funded by the National Heart, Lung, and Blood Institute of the National Institutes of Health. Its goal is to examine how health literacy and other social determinants affect the quality of care transitions from hospital to home, as well as subsequent health outcomes including medication use, functional status, health-related quality of life, unplanned health care utilization, and mortality. Moreover, we seek to determine mediators and moderators of these relationships. Some social determinants could be amenable to subsequent interventions that modify or accommodate them to improve health outcomes. Just as personalized medicine can customize health decisions based on a patient’s genetic code, healthcare and quality improvement initiatives can also be personalized based on demographic and social factors. Understanding these factors can help us not only tailor care more specifically to fit individuals’ needs and preferences, but also help improve healthcare quality and outcomes.

## Methods

### Study sample

Patient enrollment for VICS began in October 2011, and is scheduled to end in October 2015, with the goal of enrolling 3,000 patients. Adults admitted to Vanderbilt University Hospital or an affiliated community hospital, Williamson Medical Center, are eligible. Monday through Saturday, staff screen the hospital’s electronic medical records to identify patients who presented to the hospital with symptoms suggestive of ADHF and/or intermediate to high likelihood of ACS. A study investigator (hospitalist or cardiologist) confirms the diagnosis by chart review. Research assistants (RAs) then assess the presence of the following exclusion criteria: age < 18 years, inability to communicate in English, blindness, hearing impairment, lack of a working telephone, conditions that would interfere with the validity of the interview (e.g., significant dementia, active psychosis or mania), being near the end of life (hospice or home hospice), lack of cooperation, police custody, enrollment in a conflicting study, or prior enrollment in VICS. Patients who are delirious or too ill to participate early in their hospitalization, but who would be eligible otherwise, are re-assessed for up to 7 days for potential eligibility. Because many of the instruments are designed for patient self-assessment, we do not enroll surrogates to respond on the patient’s behalf. Approaching members of racial/ethnic minority groups and women is prioritized to promote their representation in the study sample.

### Study procedures

**Figure 2:**
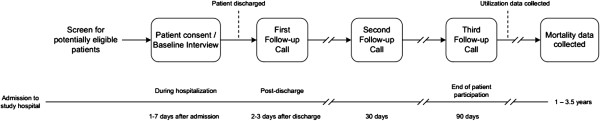
Study flow.

The VICS protocol was reviewed and approved by the Vanderbilt University Institutional Review Board. After obtaining written informed consent, RAs verbally administer the 45-minute baseline interview at the bedside. RAs have received thorough training in effective health communication, including best practices in informed consent, recruitment, and interviewing techniques. Patients are contacted by telephone for follow-up interviews at 2-3 days (range 1-7), 30 days (range 25-35), and 90 days (range 85-95) after discharge. These calls require approximately 15, 15, and 7 minutes, respectively. Additional data (e.g., measures of disease severity, comorbidities, laboratory values, verification of health care utilization) are obtained from the electronic health record and by chart abstraction. Figure 2 provides an overview of the study flow.

### Interview measures and data collection

Study data are collected and managed using the Research Electronic Data Capture (REDCap) platform [22]. REDCap is a secure, web-based application designed to support research data entry, validation, and management.

The domains assessed and their specific measures are presented and briefly described in Table 1. Data collection and analyses are grounded in the study’s conceptual framework (Figure 1).

**Table 1:** Data collection

**Domain**	**Scale**	**Description**	**Baseline**	**2-3 Days**	**30 Days**	**90 Days**
Demographics	From BRFSS [49]	Race, ethnicity, place of birth	X			
Acculturation	GBAS [23]	Language-based measure of acculturation	X			
Income/socioeconomic status		Annual household income, difficulty paying bills, employment and education status	X			
Social support	HRS, MIDUS, ESSI [33-35]	Marital status, number in household, number of close family/friends and frequency of contact, perceived social support	X		X	
Health literacy	s-TOFHLA [30]	Ability to perform basic reading tasks typical of those encountered in the health care setting	X			
Subjective health literacy	BHLS [31]	Confidence with written medical information	X			
Subjective numeracy	SNS [32]	Preferences for numerical information	X			
Cognition	SPMSQ [24]	Level of cognitive impairment	X			
Access to care		Presence of a regular health care provider	X			
Prior health care utilization		Number of hospital admissions, Emergency Department visits, and outpatient clinic visits during the last year	X			
Medication adherence	ARMS [52]	Adherence to medication regimen	X		X	
Quality of life	PROMIS* Global health status [27]	Physical functioning, pain, fatigue, emotional distress, and social health	X		X	X
Depression	PHQ-8 [47]	Presence and severity of depressive symptoms	X		X	
Vulnerable Elders Survey (≥65 yrs only)	VES-13 [28]	Health status, functional ability, and physical fitness	X		X	X
Exhaustion (≥65 yrs only)	CES-D [29]	Perception that everything is an effort, trouble getting going	X			
Health self-efficacy	PHCS [45]	Confidence in engaging in appropriate health-related behaviors	X			
Problem-solving and Decision-making	PSDM [41]	Preferences in health-related problem-solving, decision-making	X			
Diet	STC [50]	Healthy eating habits	X			
Physical activity	Exercise vital sign [51]	Exercise frequency and duration	X			
Smoking history	From BRFSS [49]	Past and current smoking status	X			
Alcohol consumption	From BRFSS [49]	Current alcohol intake	X			
Trust in health care system	RHCSDS [36]	General beliefs about the health care system	X			
Resilient coping	BRCS [46]	Tendency to cope with stress in a highly adaptive manner	X			
Religion/spirituality		Religious preference and denomination, intrinsic religious/spiritual orientation, and frequency of engaging in public or private religious/spiritual activities	X			
Electronic health literacy	eHEALS [37]	Experience using the Internet for health information	X			
Use of portals		Use of Vanderbilt online patient portal	X			
Trust in hospital providers	WFPTS [42]	Interpersonal relationship between patient and providers		X		
Quality of communication in hospital	IPC [43,44]	Quality and clarity of communication between patient and provider		X		
Preparedness for discharge	B-PREPARED [39]	Perceptions of the discharge planning process and their level of preparation		X		
Quality of the care transition	CTM-3 [40]	Care transition experiences		X		
Instrumental support	ISD	Support received with common care transition tasks		X	X	
Stress post-discharge		Stress post-discharge, contribution of financial, social/family, work, health, or other factors to stress		X		
Medication discrepancies [53]		Unintentional differences between hospital discharge regimen and what the patient thinks s/he should be taking		X		
Medication understanding [54]		Understanding of drug indications and instructions for use		X		
Outcomes: ER visits and hospitalization		Unplanned healthcare utilization after discharge			X	X
Health-related quality of life	EQ-5D [55]	Mobility, self-care, usual activities, pain, and depression			X	X
Cardiac rehabilitation		Participation in cardiac rehab, and frequency (if participating)			X	X
Outcomes: mortality		Through 1 year after last patient is enrolled and discharged				
Medical record abstraction						
Demographics		Age, gender				
Diagnosis		ACS, ADHF, or both				
Severity of illness		Including TIMI score, [25] ejection fraction				
Comorbidities		Including Elixhauser [26]				
Insurance type		Including private, Medicare, Medicaid, none, or other				
Utilization		Post-discharge ER visits and readmissions (to supplement patient-report)				

*A shortened 5-item version of the PROMIS measure is administered at baseline; the full 10-item version is administered at 30 and 90 days.

### Demographics

The baseline interview includes demographic items, contact information, employment status, educational attainment, annual household income, and marital status. Acculturation is measured using the Generic Brief Acculturation Scale (GBAS) in participants whose primary language is not English [23]. This 3-item instrument asks participants about their use of English (versus another language) to read, speak, think, and converse with friends. Cognition is assessed using the Short Portable Mental Status Questionnaire (SPMSQ), [24] a 10-item instrument that adjusts for educational level.

### Disease severity and health status

Measures of disease severity are abstracted from the patient’s chart, including: transfer from another hospital, need for intensive care, electrocardiogram (EKG) changes, ejection fraction, stent placement, bypass surgery, and the presence of shock, life-threatening arrhythmia, and cardiac arrest. Additionally, the prognostic cardiovascular risk of patients’ ACS is recorded as the clinician-reported Thrombolysis in Myocardial Infarction (TIMI) risk score [25].

Health status is represented in part by the Elixhauser index, which summarizes the presence or absence of 30 medical comorbidities [26]. Patients also report their subjective global health status at baseline through selected items pertaining to health and well-being from the NIH Patient Reported Outcomes Measurement Information System (PROMIS) [27].

Among patients aged 65 or older, vulnerability and functional status are assessed using the Vulnerable Elders Survey (VES-13), [28] a validated instrument that assesses participants’ activities and instrumental activities of daily living (ADLs and IADLs). Frailty is assessed using the exhaustion items from the Center for Epidemiologic Studies Depression Scale (CES-D), [29] self-report of unintentional weight loss prior to admission, and grip strength.

### Health literacy and numeracy

Health literacy is assessed using the short form of the Test of Functional Health Literacy in Adults (s-TOFHLA), [30] a 7-minute timed test that categorizes health literacy as inadequate, marginal, or adequate. Subjective health literacy is also assessed using the 3-item Brief Health Literacy Screen (BHLS) [31].

Numeracy, which is the ability to access, understand, and apply numerical data, is measured using a shortened 3-item version of the Subjective Numeracy Scale (SNS) [32]. This self-reported measure assesses participants’ comfort with numerical data through items about math skills and preferences for numerical information.

### Social support

Social support of friends, family, and neighborhood prior to hospitalization is assessed using measures that characterize emotional and instrumental support, as well as companionship. Items from the Health and Retirement Survey (HRS) ask participants to estimate the number of friends and family members with which they have a close relationship [33]. Additionally, items from the Midlife Development in the United States (MIDUS) survey measure frequency of contact and level of support from friends, family, and neighbors [34]. The ENRICHD Social Support Inventory (ESSI) complements these by asking about any other sources of emotional support [35].

Social support during the post-discharge period is assessed during the first follow-up call, as hospitalization may change the quality and quantity of social support received. The Instrumental Support after Discharge (ISD) survey asks about specific forms of support that participants receive with common care transition tasks, such as obtaining prescriptions, understanding the medication regimen, or transportation. Items from the MIDUS and ESSI are re-administered at the 30-day interview.

### Patient-system factors

Prior health care utilization is a strong predictor of future utilization; [9] therefore, participants are asked at baseline to report the number of hospital admissions, emergency department visits, and outpatient clinic visits during the last year prior to the index admission.

Trust in the healthcare system is measured at baseline using the Revised Health Care System Distrust Scale (RHCSDS), [36] which assesses patient perceptions of honesty, confidentiality, competence, and fidelity.

Access to care is determined by the self-reported presence of a regular health care provider at baseline. The participant’s access to and use of the Internet is assessed at baseline using items from the eHealth Literacy Scale (eHEALS) [37]. From electronic health records, the participant’s health insurance, administrative details about the index hospitalization and prior utilization, and whether the participant left the hospital against medical advice (AMA) are obtained. Leaving AMA has been shown to predict readmission, as well as higher mortality [38].

The quality of discharge planning is assessed during the post-discharge telephone call using the B-PREPARED instrument, [39] which measures participants’ perceptions of the discharge planning process and their level of preparation for performing self-care. Additionally, the quality of the care transition from hospital to home is measured using the Care Transitions Measure-3 (CTM-3) [40]. Both the B-PREPARED and CTM-3 measures have been shown to predict hospital readmission.

### Patient-provider factors

Patients’ preferences for participation in decision-making are determined using two vignettes from the Problem-Solving Decision-Making Scale (PSDM), [41] which elicits the extent to which participants prefer to share the decision-making process with their physician.

Trust in hospital providers is assessed after discharge using the Wake Forest Physician Trust Scale (WFPTS), [42] which measures the interpersonal relationship between patient and provider in domains of fidelity, competence, honesty, and overall. Given the nature of the medical teams in an academic hospital, the wording is modified to refer to all inpatient physicians who took care of them, rather than to a specific physician.

The quality of communication with inpatient providers is measured using items from the Interpersonal Processes of Care in Diverse Populations instrument (IPC), [43,44] which assesses clarity of explanations, responsiveness to patient concerns, and involvement of patients in decision-making.

### Disease self-management

Health self-efficacy is assessed using a shortened 2-item version of the Perceived Health Competence Scale (PHCS) [45]. Resilient coping, beliefs consistent with the ability to rebound from or positively adapt to significant stressors, is measured by the 4-item Brief Resilient Coping Scale (BRCS) [46]. The presence and severity of depressive symptoms at baseline is measured using the Patient Health Questionnaire (PHQ-8), [47] which is re-administered during the 30-day follow up interview, as both prevalent and incident depression are common in this setting and impact multiple outcomes [48]. Other health behaviors, such as tobacco/alcohol use, diet, and exercise are measured using the Centers for Disease Control (CDC) Behavioral Risk Factor Surveillance System (BRFSS), [49] the Starting the Conversation (STC) scale, [50] and the Exercise Vital sign, [51] respectively. Post-discharge stress is assessed as well (Table 1).

Medication adherence before hospitalization and 30 days after discharge is self-reported using a shortened 7-item version of the Adherence to Refills and Medicines Scale (ARMS) [52]. Post-discharge medication discrepancies are measured by eliciting a medication history from participants, comparing it to the list of discharge medications in the health record, and probing the reasons for any differences [53]. The RA further assesses participants’ understanding by asking them to provide the indication, dose, and frequency for representative cardiac medications [54].

### Health outcomes

General health-related quality of life, as well as functional status for all participants, is assessed using the full PROMIS 10-item measure of global physical and mental health, [27] as well as the EQ-5D [55]. Functional status for participants age 65 or older is re-assessed at 30 and 90 days post-discharge using the VES-13, described above.

Unplanned health care utilization, including emergency department visits and unplanned readmission, is assessed at 30 and 90 days after discharge from the index hospitalization, using a combination of participant report and internal as well as external medical record review.

Mortality data will be gathered for up to 3.5 years after hospital discharge, using a combination of data from the Social Security Administration, [56] medical documentation in the electronic health record, family report, and obituaries.

### Statistical analysis

The primary outcomes of interest are mortality and 30-and 90-day unplanned health care utilization. Additional outcomes include post-discharge medication discrepancies, functional status, and quality of life.

Cox proportional hazard regression models will be constructed to examine the impact of health literacy, other patient characteristics, and hospital discharge quality on unplanned health care utilization and mortality [57]. Semi-competing risk models will be used for analyses since mortality, an absorbing state, precludes readmission but not vice versa [58]. The models will include independent variables from eight broad categories that are hypothesized to influence post-discharge outcomes. They include: a) sociodemographic factors; b) health status; c) health literacy/numeracy; d) social support; e) patient-system factors; f) patient-provider factors; g) self-management; and h) care transition quality. Due to the large number of independent variables, data reduction techniques including principal component and redundancy analyses will be conducted to reduce the chance of model overfitting [59]. Smoothed Schoenfeld residuals will be used to graphically test for departure from the proportional hazards assumption [60]. With interest in understanding how health literacy may moderate the quality of discharge and outcomes among different populations, interactions between health literacy and demographic groups (e.g., age, gender, race), illness burden (e.g., Elixhauser index), and levels of social support (e.g., ESSI) will be examined. Likelihood ratio tests will be used for tests of interactions. Due to the large number of variables being collected, missing data are likely to be observed. We will use multiple imputation techniques to validly address bias and uncertainty associated with the missing data. Analyses will be performed in the R programming language (http://www.r-project.org).

In addition to the primary analyses, and guided by the conceptual framework depicted in Figure 1, path models will be constructed to explore the relationships among exogenous variables (e.g., age, health literacy, social support) and endogenous mediator (e.g., access to care, self-efficacy/health competence, resilient coping) and outcome variables (e.g., unplanned health care utilization, mortality). Model fit will be quantified with the comparative fit index, [61] the root mean square error of approximation, and the adequacy index [59]. Bootstrap-based resampling will be used to accurately summarize the potential for model overfitting and optimism.

#### Power and sample size

We will enroll 3,000 patients, all of whom will be observed for mortality until one year after the last patient is enrolled. Health care utilization and mortality data will be collected electronically and by medical record review, so we anticipate greater than 95% availability of those data. Over the course of follow-up, which will vary from one year (late enrollees) to 3.5 years (early enrollees), we anticipate the mortality rate to be approximately 23%. This is based on the assumptions that 63%, 32%, and 5% of the enrolled patients will have ACS, ADHF, and both ACS and ADHF diagnoses, respectively; one-year mortality rates will be approximately 4%, [5,6] 27%, [8] and 14%, [62] respectively; the time until death distribution will be approximately exponential; and the administrative censoring distribution (due to the end of the study) will be approximately uniform from one to 3.5 years.

Using a distribution of previously observed patients at Vanderbilt University Hospital, we anticipate participants to fall into the health literacy strata on the s-TOFHLA as follows: 10% inadequate, 9-10% marginal, and 80% adequate [53]. Assuming s-TOFHLA scores are uniformly distributed in each stratum, we conducted a simulation-based calculation to explore power as a function of s-TOFHLA effect size while adhering to the diagnostic composition (ACS, ADHF, and both) of the sample. The power to detect a 7.5%, 8.75%, and 10% drop in the hazard rate for death per 6-point increase on the s-TOFHLA scale was 80%, 90% and 96%, respectively.

### Enrollment to date

Between October 2011 and August 2013, 5350 patients were determined by clinical chart screening to have ADHF and/or intermediate to high likelihood of ACS. Some patients did not complete the full assessment of study eligibility due to logistical reasons such as rapid patient discharge (24.3%), declining to be screened (17.0%), or in-hospital death before screening was completed (1.4%). The remaining 57.3% of patients with ADHF and/or ACS completed the eligibility assessment. Among them, 50.0% were determined to be eligible, and 81.5% of eligible patients enrolled in the study. The most common reasons for ineligibility among those who completed screening were previous enrollment in VICS or a conflicting study (14.6%), or being too ill (13.8%) or too cognitively impaired (6.8%) to complete the study questionnaires.

As of August 2013, 1249 patients were enrolled. Participants had a median age of 60 at enrollment, 55.0% were male, and 59.6% were married or living with someone (Table 2). Most (62.7%) presented with ACS, while 29.7% had ADHF, and 7.6% had both conditions. In this referral center, study participants are a geographically diverse group, residing in 143 counties across 15 states. In educational attainment, 44.7% reported a high school education or less. On the s-TOFHLA, 11.8% had inadequate health literacy, and 7.4% had marginal health literacy.

**Table 2:** Participant characteristics (N = 1,249)

	
Age, median (IQR)	60 (52-69)
Male gender	687 (55.0)
Diagnosis	
ACS	783 (62.7)
ADHF	371 (29.7)
Both	95 (7.6)
Race*	
White	1029 (82.4)
Black	192 (15.4)
Other	25 (2.0)
Hispanic/latino ethnicity	24 (1.9)
Marital status	
Married/living with partner	744 (59.6)
Separated/divorced	228 (18.3)
Widowed	157 (12.6)
Single/never married	120 (9.6)
Number of people who live at home (not including patient), median (IQR)	1 (1-2)
Years of education	
0-8 (i.e. no high school)	50 (4.0)
9-11 (i.e. some high school)	127 (10.2)
12/GED	381 (30.5)
13-15 (i.e. some college)	390 (31.2)
16 (i.e. college graduate)	161 (12.9)
17+	140 (11.2)
Health literacy*	
Inadequate	148 (11.8)
Marginal	92 (7.4)
Adequate	970 (77.7)

Values are given as N (%) unless otherwise noted. IQR = interquartile range.

*Missing values: race (N = 3, 0.2%), health literacy (N = 39, 3.1%).

Completion of follow-up calls thus far demonstrates high levels of patient retention–88.2% at 2-3 days, 88.0% at 30 days, and 86.4% at 90 days after discharge.

## Discussion

The period following hospital discharge is a vulnerable time for patients, as they adjust to new medications, recover from acute illness, and cope with the challenges of acute and chronic diseases. While biomedical research has produced large advances in clinical care and the concept of personalized medicine has introduced customized pharmacology, there is an additional need to better characterize social determinants of health that influence patient outcomes. Most prior work on post-discharge outcomes has utilized administrative datasets or registries that may lack detailed information on social determinants. The Vanderbilt Inpatient Cohort Study will bring several unique contributions to the literature, including robust assessment of health literacy, social support, and other aspects of engagement with the health care system; enrollment of patients from a referral center and affiliated hospital in the South, where the burden of ACS and ADHF is high; and outcomes that include self-management, functional status, quality of life, health care utilization, and mortality. Overall, VICS seeks to identify predictors of health outcomes during this vulnerable time, determine their interrelationships, and suggest which may be suitable for intervention or customized care.

## Abbreviations

ADLs: Activities of daily living; ACS: Acute coronary syndromes; ADHF: Acute decompensated heart failure; ARMS: Adherence to refills and medicines scale; AMA: Against medical advice; BRFSS: Behavioral risk factor surveillance system; BHLS: Brief health literacy screen; BRCS: Brief resilient coping scale; CTM-3: Care transitions measure-3; CES-D: Center for epidemiologic studies depression scale; CDC: Centers for disease control; eHEALS: eHealth literacy scale; EKG: Electrocardiogram; ESSI: ENRICHD Social Support Inventory; EDW: Enterprise data warehouse; GBAS: Generic brief acculturation scale; HRS: Health and retirement survey; HCSDS: Health care system distrust scale; IADLs: Instrumental activities of daily living; ISD: Instrumental support after discharge; IPC: Interpersonal processes of care in diverse populations instrument; MIDUS: Midlife development in the United States; PHQ: Patient health questionnaire; PROMIS: Patient reported outcomes measurement information system; PHCS: Perceived health competence scale; PSDM: Problem-solving decision-making scale; RA: Research assistant; REDCap: Research electronic data capture; s-TOFHLA: Short form of the test of functional health literacy in adults; SPMSQ: Short portable mental status questionnaire; STC: Starting the conversation; SNS: Subjective numeracy scale; TIMI: Thrombolysis in myocardial infarction; VICS: Vanderbilt inpatient cohort study; VES-13: Vulnerable elders survey; WFPTS: Wake forest physician trust scale.

## Competing interests

Dr. Kripalani is a consultant to and holds equity in PictureRx, LLC, which has no role in the design or performance of this study.

## Authors’ contributions

Conception and design (AGM, AS, KAW, CC, EEV, KMG, CMD, RLR, LDC, KMD, JFS, SB, JSS, CYO, FEH, SK); acquisition, analysis, and interpretation of data (CC, KMG, JSS, SK); drafting of manuscript (AGM, AS, KAW, CC, EEV, JSS, SK); critical revision of manuscript (AGM, AS, KAW, CC, EEV, KMG, CMD, RLR, LDC, KMD, JFS, SB, JSS, CYO, FEH, SK). All authors read and approved the final manuscript.

## Pre-publication history

The pre-publication history for this paper can be accessed here:

http://www.biomedcentral.com/1472-6963/14/10/prepub
